# Satisfactory clinical and radiologic outcomes with a new shorter and modular stem for end-stage hip osteoarthritis: an international prospective multicentre pilot study

**DOI:** 10.1051/sicotj/2022005

**Published:** 2022-04-04

**Authors:** Riccardo D’Ambrosi, Aldo Toni, Jaroslaw Czubak, Jorge Guadilla, Lawrence Lieber, Ilaria Mariani, Nicola Ursino

**Affiliations:** 1 IRCCS Istituto Ortopedico Galeazzi 20161 Milano Italy; 2 IRCCS Istituto Ortopedico Rizzoli 40136 Bologna Italy; 3 SPSK im.Prof.A.Grucy 05-400 Otwock Poland; 4 HUA Santiago 01004 Vitoria Spain; 5 DuPage Medical Group Ltd., Downers Grove IL 60540 USA; 6 Institute for Maternal and Child Health – IRCCS “Burlo Garofolo” 34137 Trieste Italy

**Keywords:** Hip Arthroplasty, Multicenter Study, Short Stem, Brevius CLS, Blood Ion Levels

## Abstract

*Introduction*: This multicenter prospective cohort study aimed to assess the safety and clinical and radiologic performance of the CLS^®^ Brevius^TM^ Stem with Kinectiv^®^ Technology. *Material and Methods*: A total of 222 consecutive subjects, recruited in five different centers, qualifying for primary total hip arthroplasty (THA), were enrolled in the study. All the subjects received the CLS^®^ Brevius^TM^ Stem with Kinectiv^®^ Technology. All the enrolled study subjects underwent pre-operative clinical and radiographic evaluation. Additionally, all subjects underwent post-operative clinical, functional and radiographic evaluations at 6 months and 1, 2, 3, and 5 years. These evaluations included implant survival, pain and functional performance (Harris Hip Score [HHS], University of California, Los Angeles [UCLA] Activity Score, Oxford Hip Score), subject quality-of-life (EQ-5D), radiographic parameters, complications, and concentration of metal ions (aluminum and titanium) in blood. *Results*: No revisions were performed during the follow-up period. Of the 222 patients, only 76 completed the 5-year follow-up. Only 7 and 5 patients had aluminum and titanium 5-year evaluations, respectively. All the clinical parameters showed an overall improvement in the overtime measured with ANOVA for repeated measures; furthermore, the clinical scores showed a statistically significant improvement at 5 years with respect to pre-operative value (*p* < 0.001). Aluminum and titanium showed no variation for repeated measures at different time points (*p* > 0.05). A total of six complications were reported, of which only two were hip-related. Conclusions: The function of the CLS^®^ Brevius^TM^ Stem with Kinectiv^®^ Technology indicated that subject well-being significantly increased following THA regardless of age, gender, BMI, previous surgery, primary diagnosis, and lifestyle.

## Introduction

The CLS^®^ Brevius™ Stem with Kinectiv^®^ Technology (also referred to as CLS Brevius Kinectiv Stem) is a straight, cementless stem based on the successful anchoring philosophy of the CLS Spotorno Stem. To be more bone-conserving, the stem was distally shortened by 20% compared to the CLS Spotorno Stem. The proximal fixation principle of the CLS Spotorno Stem, with its three-dimensional taper and longitudinal ribs, demonstrated excellent clinical long-term results and remains unchanged in the CLS Brevius Kinectiv Stem. To restore individual patients’ anatomies more accurately, the CLS Brevius Stems were fused with the existing Zimmer Kinectiv Modular Neck Technology. The modularity facilitates head center restoration and, thus, soft-tissue balancing by allowing the adjustment of leg length, offset, and ante-/retroversion intraoperatively and independently from each other, without affecting proximal stem fit [1–4]. However, a modular system may lead to several complications, such as stress fractures or stem-neck dissociation, in particular during surgery [5–7]. Another major concern is related to local and systemic metal ion release [8].

The Kinectiv Technology Necks have been available on the market since 2007 with the M/L Taper Kinectiv Hip Stem, with good short-term clinical results, as reported by Duwelius et al. [9]. Comprehensive preclinical testing (including corrosion tests, fatigue tests, and pull-off tests) has been performed to ensure the mechanical and biological safety of the CLS Brevius Kinectiv Stem [10]. Currently, only one clinical study analyzes outcomes using the CLS Brevius Kinectiv Stem [11].

The primary purpose of this study was to analyze the implant survival and revision rate; the secondary aim was to evaluate clinical (pain and functional performances, subject health status) and radiographic parameters (radiolucencies, osteolysis, hypertrophy, subsidence, etc.). Finally, safety was assessed by monitoring the frequency and incidence of adverse events (AEs), serious adverse events (SAEs), adverse device effects (ADEs), and serious adverse device effects (SADEs) and analysis of metal ion concentration in the blood.

## Material and methods

### Study design

This study is a multicenter (IRCCS Istituto Ortopedico Galeazzi, IRCCS Istituto Ortopedico Rizzoli, SPSK im. Prof. A. Grucy, HUA, DuPage Medical Group Ltd), prospective, non-controlled, consecutive cohort post-market clinical follow-up study involving five orthopedic surgeons (JG, JC, AT, NU, LL) skilled in THA procedures and experienced with the implant used in this study. The Ethics Committee (EC) approval for each site was obtained for this study. The study was conducted following the STROBE checklist for cohort studies and has been registered in the Clinical Trial Registry (NCT03410940, www.clinicaltrials.gov) [12].

All the subjects signed an informed consent process, and the EC approved written informed consent prior to study enrolment. Clinical and radiological follow-up evaluations were conducted before surgery and then at 6 months from hospital discharge and at 1, 2, 3, and 5 years (± 2 months) post-surgery. Metal ion (whole blood titanium and aluminum) analysis was performed in 2 centers (IRCCS Istituto Ortopedico Galeazzi and IRCCS Istituto Ortopedico Rizzoli) pre-operatively, 6 months, 1 year, 2 years, and 5 years post-operatively. Furthermore, implant survival was calculated during the whole follow-up.

### Study population

The study population comprised a consecutive cohort of males and females who qualified for unilateral or bilateral primary THA following the inclusion criteria: minimum age of 18 years; severe hip pain and disability requiring primary unilateral or bilateral THA based on physical exam and medical history; able to cooperate in the required post-operative therapy; able to complete scheduled follow-up evaluations as described in the Informed Consent.

Exclusion Criteria: unable to give consent or to comply with the follow-up program; total prosthetic hip replacement device (including surface replacement arthroplasty, endoprosthesis, etc.) or femoral and/or acetabular osteosynthesis of the affected hip joint(s); the patient is a prisoner/mentally incompetent or unable to understand what participation in the study entails/a known alcoholic or drug abuser/anticipated to be non-compliant/pregnant; the presence of acute, chronic local or systemic infections; severe muscular, neural or vascular diseases that endanger the success of the procedure; lack of bony structures proximal or distal to the joint, so that good anchorage of the implant is unlikely or impossible; total or partial absence of the muscular or ligamentous apparatus; allergy to the implanted material, above all to metal (e.g., Vanadium); local bone tumors and/or cysts; skeletal immaturity.

A total of 222 patients were enrolled in the study, among which 106 were female (47.7%), and 116 were male (52.3%), with a mean age of 60.8 ± 13.2 years and mean BMI of 27.3 ± 4.5. Mean surgical time was 69.4 ± 19.9 min. The pre-operative diagnosis was primarily osteoarthritis in 178 patients (80.2%), avascular necrosis in 20 (9.0%), post-traumatic arthritis in 4 (1.8%), and other in 20 (9.0%). In 94 cases, a posterolateral or anterior approach was performed (42.4%), while in 34, a direct lateral approach was performed (15.2%). The detailed pre-operative and surgical data are presented in the Appendix (Tables A.1–A.12).

## Study outcome measures/endpoints

### Survivorship

The primary endpoint is defined as the implant survival (assessed by complete or partial revision of the device) [13].

### Clinical outcomes

The outcome of the treatment was assessed through the following patient-reported outcome measure scores: pain and functional performance (Harris Hip Score [HHS], University of California, Los Angeles [UCLA] Activity Score, Oxford Hip Score) [14, 15] and subject quality-of-life (EQ-5D) [16]

### Radiographic assessment

Radiographic assessment was performed in each center by a skilled musculoskeletal radiologist and evaluated in each patient restoration of anatomy, radiolucencies, osteolysis, hypertrophy, subsidence, and bone stock changes [17].

### Adverse events

Adverse events (AEs) were defined as an undesirable clinical development in a participant who was not present at baseline or increased in severity after treatment. AEs were assessed and graded in regards to severity:

Mild: awareness of a sign or symptom which does not interfere with the participants’ usual daily activity and/or is transient, resolving with the use of simple interventions, including simple analgesia.
Moderate: interferes with the participants’ usual daily activity and/or requires symptomatic treatment, including regular analgesia (i.e., opioid analgesia).
Severe: symptom(s) causing severe discomfort with significant impact on the participants’ usual daily activity.
Serious: an unexpected medical incident which requires hospitalization, results in long-term disability, is life-threatening, or results in death [18].

Furthermore, metal ion concentration in the blood was measured.

### Statistical analysis

The mean of clinical scores (UCLA, EQ-5D, HHS, OXFORD) and laboratory findings (aluminum and titanium) were compared at different time assessments with a repeated measure analysis of variance. A Toeplitz, autoregressive, or unstructured covariance matrix within the subject residuals was selected according to how well of a fit it was with the model (Akaike Information Criterion). The Wilcoxon signed-rank test was preferred in case of deviation from the assumptions of the model.

A secondary analysis was conducted to assess the variations by gender, age, BMI, primary diagnosis (osteoarthritis vs. others), tobacco and alcohol consumption, femoral size, taper (straight vs. other), previous surgery, and *surgical approach* (posterolateral vs. anterolateral vs. direct lateral). The age, BMI, and femoral size were included in the repeated measure model as binary variables dichotomized at their average rounded value. When a non-parametric test was preferred, the Kruskal–Wallis and Wilcoxon–Mann–Whitney tests were performed to compare means of different groups at the same time period (within time comparison), while the Wilcoxon signed-rank test was performed to compare means of the same group at different time periods (within-group comparison). Furthermore, the correlation among variables was calculated and tested using the Pearson or Spearman rank correlation according to the distribution of the variables. Finally, the proportions of radiographic *diagnosis/results* at different time assessments were compared with a Fisher exact test or a Chi-squared test.

The Bonferroni correction was used for multiple comparisons. A two-tailed *p*-value less than 0.05 was considered statistically significant. The statistical analyses were performed using SAS 9.4.

## Results

### Implant survival

No revisions were performed during the follow-up period.

### Clinical outcomes

All the clinical parameters showed overall improvement over time measured with ANOVA for repeated measures; furthermore, clinical scores showed a statistically significant improvement at 5 years with respect to pre-operative value (*p* < 0.001). The detailed results are reported in Table 1.

**Table 1:** Clinical evaluation at each time point. Multicomparison tests were performed with the analysis of variance (ANOVA) test for repeated measures, while difference between time points was performed with Bonferroni adjusted *p*-value.

	Preop	6 Month	1 Year	2 Year	3 Year	5 Year
Score						
UCLA*	3.8 ± 1.6 (222)	5.5 ± 1.4^a,b^ (195)	5.9 ± 1.6^a,b^ (178)	6.1 ± 1.6^a,b^ (145)	6.2 ± 1.6^a,b^ (143)	6.4 ± 1.4^a,b^ (76)
EQ5D*	0.5 ± 0.3 (222)	0.9 ± 0.2^a^ (193)	0.9 ± 0.2^a,b^ (176)	0.9 ± 0.2^a^ (144)	0.9 ± 0.2^a^ (143)	0.9 ± 0.1^a,b^ (73)
HHS*	49.5 ± 12.9 (222)	93.8 ± 8.9^a,b^ (194)	96.3 ± 6.6^a,b^ (177)	96.3 ± 7.1^a,b^ (144)	96.9 ± 7.1^a,b^ (142)	97.3 ± 4.7^a,b^ (73)
Oxford*	22.1 ± 7.6 (222)	43.4 ± 5.4^a,b^ (195)	44.8 ± 4.8^a,b^ (178)	45.3 ± 4.3^a,b^ (145)	45.8 ± 4.0^a,b,c^ (142)	46.4 ± 2.6^a,b^ (76)
Laboratory findings						
Aluminum (μg/L)	12.1 ± 1.8 (31)	12.2 ± 1.8 (25)	14.2 ± 3.6 (24)	13.8 ± 3.7 (21)	–	14.1 ± 2.5 (7)
Titanium (μg/L)	2.1 ± 0.7 (9)	2.4 ± 1.7 (43)	2.5 ± 1.8 (35)	2.9 ± 2.5 (35)	–	1.3 ± 0.3 (5)

Scores and laboratory findings are presented as mean ± SD (*n*).

* Significant difference (*p* < 0.05) by time; – = not evaluated.

a Statistical significant difference versus Preop value.

b Statistical significant difference versus 6-month value.

c=b Statistical significant difference versus 1-year value.

### Radiographic results

**Figure 1:**
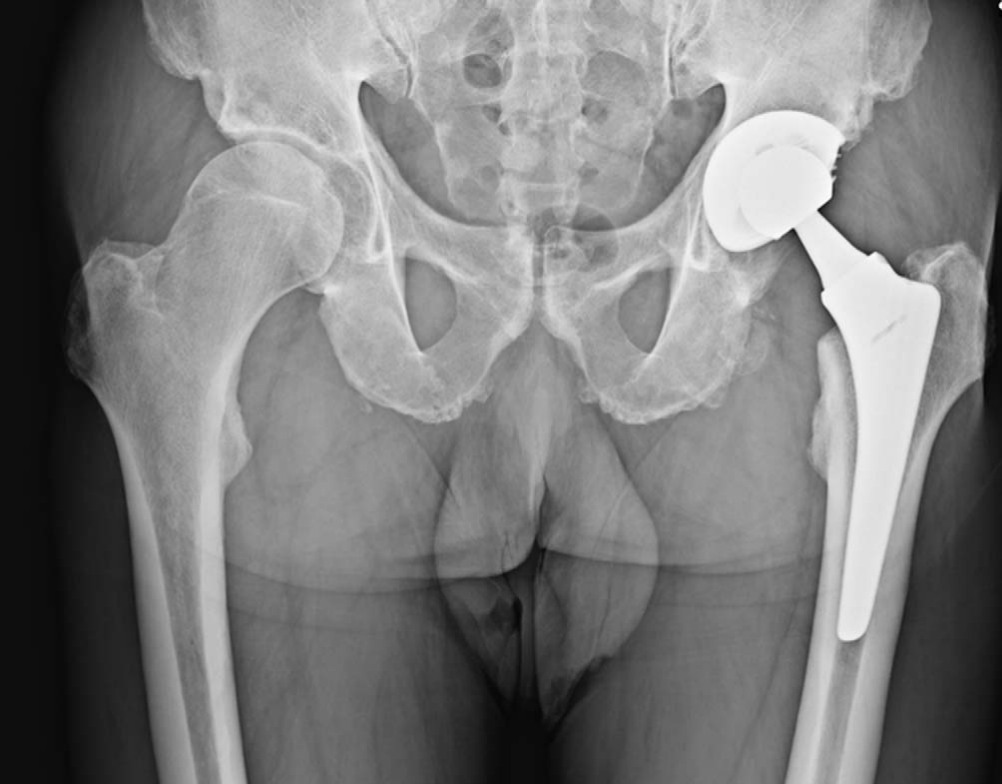
Anteroposterior pelvis X-ray showing CLS Brevius Kinectiv Stem 5 years after surgery with no signs of mobilization or osteolysis.

The only significant radiographic changes were regarding the percentage of patients with acetabular radiolucency at 3 and 5 years (*p* < 0.05). The detailed results are reported in Table 2. Figure 1 shows radiographic results at 5-year follow-up with no signs of mobilization or osteolysis.

**Table 2:** Radiographic assessment at each follow-up; Comparison by time assessment was performed using Bonferroni adjusted *p*-value.

	Radiographic Assessment		
Follow up visit	Yes	No	Total
	*n* (%)	*n* (%)	*N* (%)
Anteroposterior Femoral Periosteal Cortical Hypertrophy			
6 Month	1 (0.5%)	191 (99.5%)	192 (100.0%)
1 Year	1 (0.6%)	176 (99.4%)	177 (100.0%)
2 Year	5 (3.5%)	138 (96.5%)	143 (100.0%)
3 Year	5 (3.5%)	136 (96.5%)	141 (100.0%)
5 Year	4 (5.3%)	72 (94.7%)	76 (100.0%)
Lateral Femoral Periosteal Cortical Hypertrophy			
6 Month	0	192 (100.0%)	192 (100.0%)
1 Year	0	177 (100.0%)	177 (100.0%)
2 Year	2 (1.4%)	141 (98.6%)	143 (100.0%)
3 Year	3 (2.1%)	138 (97.9%)	141 (100.0%)
5 Year	3 (3.9%)	73 (96.1%)	76 (100.0%)
Anteroposterior Femoral Bone Condensation			
6 Month	0	192 (100.0%)	192 (100.0%)
1 Year	4 (2.3%)	173 (97.7%)	177 (100.0%)
2 Year	3 (2.1%)	140 (97.9%)	143 (100.0%)
3 Year	3 (2.1%)	138 (97.9%)	141 (100.0%)
5 Year	4 (5.3%)	72 (94.7%)	76 (100.0%)
Lateral Femoral Bone Condensation			
6 Month	0	192 (100.0%)	192 (100.0%)
1 Year	1 (0.6%)	176 (99.4%)	177 (100.0%)
2 Year	2 (1.4%)	141 (98.6%)	143 (100.0%)
3 Year	2 (1.4%)	139 (98.6%)	141 (100.0%)
5 Year	3 (3.9%)	73 (96.1%)	76 (100.0%)
Anteroposterior Femoral Sclerotic Halo for zone			
6 Month	0	192 (100.0%)	192 (100.0%)
1 Year	2 (1.1%)	175 (98.9%)	177 (100.0%)
2 Year	0	143 (100.0%)	143 (100.0%)
3 Year	1 (0.7%)	140 (99.3%)	141 (100.0%)
5 Year	1 (1.3%)	75 (98.7%)	76 (100.0%)
Anteroposterior Acetabular Radiolucency			
6 Month	6 (3.1%)	186 (96.9%)	192 (100.0%)
1 Year	7 (4.0%)	170 (96.0%)	177 (100.0%)
2 Year	7 (4.9%)	136 (95.1%)	143 (100.0%)
3 Year	8^a,b,c^ (5.7%)	133^a,b,c,^ (94.3%)	141 (100.0%)
5 Year	8^d^ (10.5%)	68^d^ (89.5%)	76 (100.0%)
Heterotopic Ossification			
6 Month	12 (6.3%)	180 (93.8%)	192 (100.0%)
1 Year	24 (13.6%)	153 (86.4%)	177 (100.0%)
2 Year	27^a^ (18.9%)	116^a^ (81.1%)	143 (100.0%)
3 Year	22 (15.6%)	119 (84.4%)	141 (100.0%)
5 Year	13 (17.1%)	63 (82.9%)	76 (100.0%)
Anteroposterior Femoral Radiolunceny			
6 Month	4 (2.1%)	188 (97.9%)	192 (100.0%)
1 Year	6 (3.4%)	171 (96.6%)	177 (100.0%)
2 Year	6 (4.2%)	137 (95.8%)	143 (100.0%)
3 Year	5 (3.5%)	136 (96.5%)	141 (100.0%)
5 Year	6 (7.9%)	70 (92.1%)	76 (100.0%)

* Significant difference (*p* < 0.05) by time; – = not evaluated.

a Statistical significant difference versus 6-month value.

b Statistical significant difference versus 1-year value.

c Statistical significant difference versus 2-year value.

d Statistical significant difference versus 3-year value.

## Subgroup analyses

### Gender

Both male and female groups showed significant improvement in all clinical scores and their respective pre-operative value (*p* < 0.05). The female group showed a lower UCLA and EQ-5D score with respect to the male group at the pre-operative visit and at 6 months and 5-year follow-up (*p* < 0.05). For the HHS score, the female group showed lower value at pre-operative follow-up and at 6 months and 1 year (*p* < 0.05), while for the Oxford score, the female group showed lower scores at pre-operative follow-up and then at 6 months, 1 and 2-year follow-up. No difference among groups was noted regarding aluminum and titanium ion levels. The detailed results are reported in Table 3.

**Table 3:** Subgroup analysis by gender; difference between time point was performed with Bonferroni adjusted *p*-value.

Time Point	Mean value and comparison within time period		
	Male	Female	
	Unadjusted mean ± SD (*n*)	Unadjusted mean ± SD (*n*)	*p*-value
UCLA			
Preop	4.2 ± 1.7 (116)	3.5 ± 1.4 (106)	0.0013*
6 Month	5.8 ± 1.4^a^ (97)	5.2 ± 1.4^a^ (98)	0.0033*
1 Year	6.0 ± 1.6^a^ (91)	5.8 ± 1.6^a^ (87)	0.4767
2 Year	6.2 ± 1.8^a^ (73)	5.9 ± 1.4^a,b^ (72)	0.1968
3 Year	6.3 ± 1.8^a,b^ (76)	6.1 ± 1.4^a,b^ (67)	0.2608
5 Year	6.9 ± 1.5^a^ (34)	6.1 ± 1.3^a^ (42)	0.0142*
EQ5D			
Preop	0.5 ± 0.2 (116)	0.4 ± 0.3 (106)	0.0015*
6 Month	0.9 ± 0.1^a^ (96)	0.8 ± 0.2^a,b^ (97)	0.0104*
1 Year	0.9 ± 0.1^a^ (89)	0.9 ± 0.2^a^ (87)	0.1083
2 Year	0.9 ± 0.2^a^ (73)	0.9 ± 0.2^a^ (71)	0.1205
3 Year	0.9 ± 0.2^a^ (76)	0.9 ± 0.2^a^ (67)	0.2762
5 Year	1.0 ± 0.1^a,b^ (34)	0.9 ± 0.1^a,b^ (39)	0.0423*
HHS			
Preop	52.3 ± 11.7 (116)	46.4 ± 13.4 (106)	0.0010*
6 Month	96.2 ± 6.3^a^ (98)	91.3 ± 10.4^a^ (96)	0.0006*
1 Year	98.1 ± 3.3^a,b^ (91)	94.4 ± 8.5^a,b^ (89)	0.0010*
2 Year	97.1 ± 6.7^a,b^ (73)	95.6 ± 7.5^a,b^ (71)	0.0155
3 Year	96.6 ± 8.3^a^ (76)	97.3 ± 5.5^a,b^ (66)	0.4177
5 Year	97.7 ± 4.3^a,b^ (34)	96.9 ± 5.0^a,b^ (39)	0.3573
Oxford			
Preop	24.0 ± 7.5 (116)	20.0 ± 7.2 (106)	0.0002*
6 Month	44.7 ± 4.2^a^ (97)	42.0 ± 6.1^a^ (98)	0.0006*
1 Year	45.7 ± 3.5^a^ (91)	43.9 ± 5.8^a,b^ (87)	0.0420*
2 Year	45.8 ± 4.2^a,b^ (73)	44.8 ± 4.3^a,b^ (72)	0.0060*
3 Year	45.6 ± 4.9^a,b^ (76)	46.1 ± 2.8^a,b,b^ (66)	0.8770
5 Year	46.7 ± 2.8^a,b^ (34)	46.2 ± 2.5^a,b^ (42)	0.1224
Aluminum (μg/L)			
Preop	11.8 ± 1.4 (22)	12.8 ± 2.5 (9)	0.2861
6 Month	12.4 ± 1.7 (13)	12.0 ± 1.9 (12)	0.3986
1 Year	14.6 ± 3.9 (17)	13.3 ± 3.1 (7)	0.3730
2 Year	14.3 ± 4.2 (14)	12.6 ± 2.3 (7)	0.2960
5 Year	13.0 ± 2.7 (4)	15.6 ± 1.5 (3)	0.1573
Titanium (μg/L)			
Preop	2.5 ± 0.7 (4)	1.9 ± 0.7 (5)	0.2207
6 Month	2.9 ± 2.2 (22)	1.8 ± 0.7 (21)	0.0800
1 Year	2.9 ± 2.2 (21)	1.9 ± 0.6 (14)	0.4082
2 Year	3.2 ± 2.9 (18)	2.3 ± 0.9 (9)	0.5707
5 Year	1.4 ± 0.3 (3)	1.2 ± 0.2 (2)	0.2482

Scores and laboratory findings are presented as mean ± SD (*n*).

* Significant difference (*p* < 0.05) by time; – = not evaluated.

a Statistical significant difference versus Preop value.

b Statistical significant difference versus 6-month value.

c=b Statistical significant difference versus 1-year value.

### Age

Analysis by age showed a significant improvement in all clinical scores with respect to the pre-operative value for both groups (< 60 years and ≥ 60 years) (*p* < 0.05). Furthermore, the older group showed lower UCLA scores at 1, 2, and 3 years (*p* < 0.05), lower HHS at 3 years, and lower Oxford scores at 1 and 3 years. The titanium ion levels were lower in the older group at pre-operative evaluation (*p* < 0.05). The detailed results are reported in Table 4.

**Table 4:** Subgroup analysis by age; difference between time point was performed with Bonferroni adjusted *p*-value.

Follow up visit	Mean value and comparison within time period		
	<60 yrs	≥60 yrs	
	Unadjusted mean ± SD (*n*)	Unadjusted mean ± SD (*n*)	*p*-value
UCLA			
Preop	3.8 ± 1.6 (84)	3.9 ± 1.6 (138)	0.7022
6 Month	5.7 ± 1.4^a^ (70)	5.4 ± 1.4^a^ (125)	0.1219
1 Year	6.5 ± 1.3^a,b^ (68)	5.5 ± 1.6^a^ (110)	<.0001*
2 Year	6.7 ± 1.3^a,b^ (58)	5.6 ± 1.6^a^ (87)	<.0001*
3 Year	6.8 ± 1.4^a,b^ (59)	5.8 ± 1.6^a^ (84)	<.0001*
5 Year	6.8 ± 1.2^a^ (33)	6.2 ± 1.5^a^ (42)	0.0724
EQ5D			
Preop	0.4 ± 0.3 (84)	0.5 ± 0.3 (138)	0.0525
6 Month	0.9 ± 0.2^a^ (71)	0.9 ± 0.1^a^ (122)	0.8100
1 Year	0.9 ± 0.2^a,b^ (67)	0.9 ± 0.2^a^ (109)	0.3107
2 Year	0.9 ± 0.2^a,b^ (58)	0.9 ± 0.2^a^ (86)	0.8005
3 Year	0.9 ± 0.1^a,b^ (59)	0.9 ± 0.2^a^ (84)	0.1067
5 Year	1.0 ± 0.1^a,b^ (30)	0.9 ± 0.1^a,b^ (43)	0.2594
HHS			
Preop	48.6 ± 12.1 (84)	46.4 ± 13.3 (138)	0.4109
6 Month	94.2 ± 8.7^a^ (70)	91.3 ± 9.0^a^ (124)	0.4148
1 Year	97.5 ± 4.5^a,b^ (68)	94.4 ± 7.6^a,b^ (109)	0.0588
2 Year	97.5 ± 4.1^a,b^ (58)	95.6 ± 8.5^a,b^ (86)	0.1517
3 Year	98.4 ± 5.0^a,b^ (59)	97.3 ± 8.2^a,b^ (83)	0.0383*
5 Year	98.7 ± 2.4^a,b^ (30)	96.9 ± 5.6^a,b^ (43)	0.0842
Oxford			
Preop	21.6 ± 7.7 (84)	22.3 ± 7.6 (138)	0.3280
6 Month	43.5 ± 5.8^a^ (71)	43.3 ± 5.3^a^ (124)	0.2798
1 Year	46.1 ± 3.1^a,b^ (68)	44.0 ± 5.5^a^ (110)	0.0057*
2 Year	46.2 ± 2.6^a,b^ (58)	44.7 ± 5.0^a,b^ (87)	0.1743
3 Year	46.7 ± 2.8^a,b^ (59)	45.2 ± 4.6^a,b^ (83)	0.0114*
5 Year	47.1 ± 1.5^a,b^ (33)	45.9 ± 3.2^a,b^ (43)	0.0524
Aluminum (μg/L)			
Preop	12.1 ± 2.5 (11)	12.1 ± 1.4 (20)	0.2648
6 Month	12.3 ± 2.0 (13)	12.0 ± 1.5 (12)	0.7234
1 Year	14.0 ± 2.9 (13)	14.4 ± 4.5 (11)	0.8846
2 Year	15.1 ± 4.4 (12)	12.0 ± 1.0 (9)	0.1019
5 Year	13.5 ± 3.0 (3)	14.6 ± 2.3 (4)	0.4795
Titanium (μg/L)			
Preop	2.4 ± 0.5 (7)	1.2 ± 0.2 (2)	0.0404*
6 Month	2.4 ± 1.4 (19)	2.4 ± 2.0 (24)	0.4123
1 Year	2.6 ± 1.6 (17)	2.4 ± 2.0 (18)	0.4976
2 Year	3.3 ± 3.3 (14)	2.4 ± 0.8 (13)	0.7891
5 Year	1.4 ± 0.4 (2)	1.2 ± 0.2 (3)	0.5637

Scores and laboratory findings are presented as mean ± SD (*n*).

* Significant difference (*p* < 0.05) by time; – = not evaluated.

### BMI

According to the BMI analysis, significant improvement was shown in all clinical scores with respect to the pre-operative value for both groups (BMI < 27 and BMI ≥ 27) (*p* < 0.05). The only clinical difference was noted for the UCLA score at a 2-year follow-up (*p* < 0.05). The detailed results are reported in the Appendix (Tables A.1–A.12).

### Primary diagnosis

Analysis by primary diagnosis showed significant improvement in all the clinical scores with respect to the pre-operative value for both groups (*p* < 0.05). Furthermore, the patients with primary osteoarthritis showed higher pre-operative values for UCLA, EQ-5D, Oxford and HHS (*p* < 0.05). A higher value of Oxford score was also found at a 3-year follow-up (*p* < 0.05). The detailed results are reported in the Appendix (Tables A.1–A.12).

### Tobacco and alcohol consumption

Both tobacco and alcohol consumers showed an overall improvement in all clinical scores with respect to the pre-operative value (*p* < 0.05). No other statistically significant differences with non-consumers were noted (*p* > 0.05). The detailed results are reported in the Appendix (Tables A.1–A.12).

### Femoral size

Both groups divided based on femoral size (femoral size < 10 and femoral size ≥ 10) showed an overall improvement in all the clinical scores with respect to the pre-operative value (*p* < 0.05). The detailed results are reported in the Appendix (Tables A.1–A.12).

### Previous surgery

The patients with previous hip surgery reported similar results as the patients who underwent surgery for the first time and showed an overall improvement in all clinical scores with respect to the pre-operative value (*p* < 0.05). The detailed results are reported in the Appendix (Tables A.1–A.12).

### Complications

Only two hip-related complications were reported: in one case, hip pain, swelling, and wound redness after 3 weeks of the index procedure, while in the other case, psoas tendonitis was resolved with one corticosteroid shot. Aluminum and titanium showed no variation for repeated measures at different time points (*p* > 0.05). No revisions were performed during the follow-up period.

### Correlations

The significant correlations with surgical time are reported in Table 5. The significant correlation between socio-demographic, surgical characteristics data, clinical scores, and laboratory values are reported in the Appendix (Tables A.1–A.12).

**Table 5:** Significant correlations between surgical time and blood loss, clinical scores, and laboratory values.

Variables correlated with surgical time	Rho	*p*-value	*N*	
Blood loss	0.69899	<.0001*	222	
Clinical scores				
	1 Year	0.15	0.0423*	178
	5 Year	−0.25	0.0322*	76
EQ5D	Preop	−0.25	0.0002*	222
HHS	Preop	−0.24	0.0003*	222
	6 Month	−0.36	<.0001*	194
	1 Year	−0.26	0.0005*	177
	2 Year	−0.34	<.0001*	144
	3 Year	−0.26	0.0015*	142
	6 Month	−0.20	0.0051*	195

*Significant difference (*p* < 0.05).

## Discussion

To our knowledge, this is the first and largest multicentric short- to mid-term follow-up study on the CLS Brevius Kinectiv Stem, with 222 implants stratified by age, gender, BMI, primary diagnosis, previous surgery, and lifestyle. Our results demonstrated excellent implant survivorship with no revision surgeries; overall, the patients demonstrated statistically and clinically significant improvements in all clinical parameters (Harris Hip Score, Oxford Hip Score, UCLA, and EQ-5D) as well as improvements in multiple radiographic measurements at the final follow-up. There are several limitations to this study: first of all, the short- to mid-term follow-up, and, furthermore, the lack of a control group to assess clinical and radiological differences analyzing alternative arthroplasty implants. Furthermore, at a 5-year follow-up, many patients were lost. Another limitation of the study is the lack of specifying the previous surgery performed in some patients, but this was indifferent, as between the two groups, there were no differences in all follow-up. This finding is also confirmed by the literature, which shows that after several types of surgery, the clinical results for hip replacement are excellent [19–21].

Another limitation of the study is the different surgical approaches used, but also, in this case, the literature shows that the approach does not affect the final result therefore, it will not be a bias for our study [22–25].

Currently, no multi-centre study has been performed regarding the clinical and radiological results of CLS Brevius Kinectiv Stem; multi-centre collaboration can result in higher rates of patient enrolment than single-center trials, thereby generating larger studies of shorter duration. A contrasting result that emerged from the study is the low number of complications reported during the follow-up study. This can be due to the multicentric nature of the study, where the enrolment of patients in several sites enhances the generalizability of the results to similar patients in similar settings. However, at the same time, the wide variation in organizational issues among sites may influence patient outcomes [26–28], limiting the extrapolation of the results of multicenter studies to other patients with different case mixes. In multicenter studies, a rigorous protocol is used to ensure uniform data collection; however, heterogeneity in clinical practice among different centers may be a major confounding factor in interpreting the results of these studies. Despite the possible bias of a multicenter study and the different experiences of individual surgeons, the results reported were similar, ranging from good to excellent. In this scenario, to avoid mistakes resulting from the differences in the surgeons’ experiences, a key role is played by the learning curve. In fact, different approaches have been taken by different surgeons, creating a possible bias. In 2016, den Hartog et al. evaluated the literature regarding the anterior approach in comparison to other approaches, investigating if there is a learning curve for the anterior approach [29]. There was strong evidence that showed no difference in component placement between the anterior approach and other approaches. Also, strong evidence for faster post-operative recovery and less need for assistive devices after the anterior approach was found. All the other studied parameters demonstrated conflicting evidence. Although the learning curve for the anterior approach is not yet clear, this learning curve should not be neglected.

Similarly, Padilla et al. evaluated the learning curve among the THA recipients using a novel short-stem hip prosthesis, concluding that this stem model is a safe alternative for THA, reporting a fracture incidence of 2.9% among patients [30]. However, surgeons should remain cautious when utilizing new implant systems and expect a learning curve estimated at 30 cases.

Currently, only one study in the literature reported clinical results with this stem, reporting a series of 155 patients at a mean follow-up of 32 months. The mean HHS, it was reported, improved from 32 points pre-operatively to 92 points at the final follow-up, while the stem survival rate was 99.4%. Overall, the results were excellent in 148 hips (87%), good in 14 hips (8.2%), fair in six hips (3.6%), and poor in two hips (1.2%). The intraoperative complications included a calcar fissure in three hips (1.7%). The correct femoral offset was reproduced in 97%, while the planned center of hip rotation was achieved in 98%. Only one hip underwent early stem revision; this was due to major subsidence [11].

Analyzing the results from the study of Graceffa et al. [11], we can confirm good to excellent results in almost all patients with a high rate of survival of the implant.

Despite these excellent outcomes, the major limitation of the CLS Spotorno Stem was that it had only three possible femoral-neck inclination angles (145°, 135°, and 125°) and five different lengths of the metallic femoral heads (−3.5 mm; 0 mm, +3.5 mm, 7 mm, and 10 mm); multiple combinations of these parameters allowed the correct center of hip rotation to be reproduced in many, but not all, cases [31–33].

However, the addition of a further interface (the modular femoral neck) might potentially be associated with complications such as stress fractures caused by corrosion [5, 34, 35] or stem-neck disassociation, in particular during dislocation reduction maneuvers [6]. Another major concern is the correlation between increased modularity and corrosion associated with titanium ion release under in vivo conditions [7], leading to local tissue infiltration and adverse periprosthetic tissue reactions [8]. For this reason, we decided to evaluate titanium and aluminum blood ion levels over time, and they also showed no significant increase at the 5-year follow-up.

The release of ions in the blood remains a big challenge for all hip surgeons. In 2013, Catalani et al. verified the correlation of vanadium levels among different matrices and assessed reference levels of the ion in a population of patients wearing well-functioning hip prostheses, they observed that the values in the serum were above the upper limit of the reference values in 42% of patients (29% in urine and 13% in whole blood) [36].

Additionally, Bistolfi et al. tried to establish if an increase in surface area can lead to a significant increase in systemic metal levels. Patients with trabecular titanium did not have significantly higher metal ion levels than patients with conventional cups for up to 2 years. A trend over time was statistically significant in blood and urine for aluminum and titanium concentrations [37].

These results appear in contrast with blood metal levels analyzed in our study, where no significant increase of aluminum and titanium was found in the entire follow-up; furthermore, no significant correlations were found between metal ions and complications or clinical scores, confirming the safety of the CLS Brevius Kinectiv Stem implant.

Analyzing the correlations, we found that several parameters directly correlated with surgical time, in particular blood loss and clinical scores. The operative time has frequently been implicated as a risk factor for complications, including infection, venous thromboembolism, and neurologic deficit after arthroplasty, and it remains a potentially modifiable variable that is of interest to surgeons and hospitals interested in quality improvement [38–41]. These findings have been confirmed by Duchman et al., who analyzed the American College of Surgeons National Surgical Quality Improvement Program data. The database queried from 2011 to 2013 demonstrated overall complications increasing in patients whose operative time was > 120 min (5.9%) compared to patients whose operative time < 60 min or 60–120 min (4.6% and 4.8%, respectively; *p* < 0.001). Wound complications, including surgical site infection, were also increased for procedures lasting >120 min. In a multivariable analysis, operative time exceeding 120 min remained an independent predictor of any complication and wound complication, with each 30-minute increase in operative time beyond 120 min further increasing risk. Patient age < 65 years, sex male, race black, body mass index > 30 kg/m^2^, and an American Society of Anesthesiologists classification of 3 or 4; predicted operative time > 120 min [42].

Surace et al. confirmed these data, reporting a strong correlation between increased operative time and perioperative complications. Additionally, this study suggests an optimal time of approximately 80 min as a goal for surgeons, which may be associated with less risk of complications following THA [43].

Finally, radiograph findings showed a change for acetabular radiolucency only at 3 and 5 years. A thin (< 2 mm), isolated radiolucent band around the rough surface of an uncemented component, frequently well delineated by a thin sclerotic margin, non-progressive after 2 years, can be considered normal, as it indicates fibrous ingrowth and is thought to provide sufficient stability [44].

## Conclusions

The functional outcomes measured in this first prospective international multicenter study of the CLS Brevius Kinectiv Stem indicated that subject’s well-being significantly increased following THA regardless of age, gender, BMI, previous surgery, primary diagnosis, and lifestyle. Pain, functional measures, and health status exhibited statistically significant improvement maintained through the entire follow-up. The radiographic parameters also presented a low incidence of findings. Titanium and aluminum blood levels did not show a significant increase over time. Overall, the treatment of primary or secondary hip osteoarthritis using the CLS Brevius Kinectiv Stem resulted in reliable functional and radiological outcome improvement at short-term follow-up in this series.

## Competing interests

The authors declare that they have no conflict of interest.

## Funding

This study was funded by Zimmer-Biomet.

## Ethical approval

The study received ethical approval, and the file is attached to the submission.

## Consent to participate

Informed consent was obtained from all individual participants included in the study.

## Consent to publish

Informed consent was obtained from all individual participants to publish anonymously the data.

## Authors’ contributions

All authors contributed equally.

## Availability of data and materials

Data are available under request to Zimmer-Biomet.

Study Registration: Clinical Trial Registry – NCT03410940; www.clinicaltrials.gov.

## Appendix

**Table A.1.** Different femoral head used.

Femoral Head	*N*	%
Biolox Delta Ceramic	169	76.1%
Centerpulse CoCR	12	5.4%
Cerasul Ceramic	4	1.8%
Protasul 12/14 Neck Taper	7	3.2%
Sulox Ceramic, 12/14 Taper	3	1.4%
VerSys CoCR 12/14 Taper	26	11.7%
Not available	1	0.4%

**Table A.2.** Acetabular cup used.

Acetabular Cup	*N*	%
ALLOCLASSIC VARIALL UNCEMENTED	14	6.3%
Allofit IT, Protasul	8	3.6%
Allofit IT, Protasul with Screw Holes	2	0.9%
Allofit Uncemented	33	14.8%
Allofit Uncemented with Screw Holes	25	11.3%
Continuum TM CLUSTER HOLES POROUS	89	40.2%
Continuum TM MULTI HOLES POROUS	2	0.9%
TM Modular, Cluster-Holed	8	3.6%
Trilogy, Cluster-Holed, F/M	40	18.0%
Not Available	1	0.4%

**Table A.3.** Acetabular liner used.

Alpha Durasul, Hooded	22	9.9%
Alpha Durasul, Standard	36	16.2%
Biolox delta Taper Liner	45	20.4%
Gamma Cerasul Insert	1	0.4%
Gamma Durasul Insert	8	3.6%
Gamma PE Insert	5	2.3%
LONGEVITY IT, XLPE, Elevated Rim	1	0.4%
LONGEVITY IT, XLPE, Neutral	4	1.8%
LONGEVITY IT, XLPE, Offset	2	0.9%
Trilogy Longevity, XLPE, 3.5 mm Offset	17	7.7%
Trilogy Longevity, XLPE, 3.5 mm Offset, 10 Degree Elevated Rim	31	13.9%
VIVACIT-E NEUTRAL	49	22.1%
Not available	1	0.4%

**Table A.4.** Kinectiv neck offset used.

OFFSET	*N*	%
EXT	80	36.0%
RED	15	6.7%
STD	111	50.0%
XEXT	14	6.3%
XRED	1	0.5%
Not available	1	0.5%

**Table A.5.** Kinectiv neck version.

Version	*N*	%
Anteverted	15	6.8%
Retroverted	11	4.9%
Straight	195	87.8%
Not available	1	0.5%

**Table A.6.** Subgroup analysis by BMI.

Follow up visit	Mean value and comparison within time period			Comparison by time within subgroups (Bonferroni adjusted *p*-value)									
	BMI < 27	BMI ≥ 27		BMI < 27					BMI ≥ 27				
	Unadjusted mean ± SD (*n*)	Unadjusted mean ± SD (*n*)	*p*-value	Preop	6 Month	1 Year	2 Year	3 Year	Preop	6 Month	1 Year	2 Year	3 Year
UCLA													
Preop	3.9 ± 1.6 (113)	3.8 ± 1.6 (109)	0.4800	–	–	–	–	–	–	–	–	–	–
6 Month	3.5 ± 1.4 (101)	5.5 ± 1.4 (94)	0.9481	<.0001*	–	–	–	–	<.0001*	–	–	–	–
1 Year	6.1 ± 1.6 (86)	5.7 ± 1.6 (92)	0.0531	<.0001*	0.0054*	–	–	–	<.0001*	1.0000	–	–	–
2 Year	6.4 ± 1.6 (73)	5.8 ± 1.5 (72)	0.0078*	<.0001*	<.0001*	1.0000	–	–	<.0001*	1.0000	1.0000	–	–
3 Year	6.4 ± 1.6 (71)	6.0 ± 1.5 (72)	0.0876	<.0001*	<.0001*	1.0000	1.0000	–	<.0001*	1.0000	1.0000	1.0000	–
5 Year	6.7 ± 1.3 (42)	6.1 ± 1.5 (34)	0.0895	<.0001*	0.0020*	1.0000	1.0000	1.0000	<.0001*	1.0000	1.0000	1.0000	1.0000
EQ5D													
Preop	0.5 ± 0.3 (113)	0.4 ± 0.3 (109)	0.3586	–	–	–	–	–	–	–	–	–	–
6 Month	0.9 ± 0.2 (101)	0.9 ± 0.2 (92)	0.3175	<.0001*	–	–	–	–	<.0001*	–	–	–	–
1 Year	0.9 ± 0.2 (86)	0.9 ± 0.2 (90)	0.2969	<.0001*	0.0872	–	–	–	<.0001*	1.0000	–	–	–
2 Year	0.9 ± 0.2 (72)	0.9 ± 0.2 (72)	0.2811	<.0001*	0.1004	1.0000	–	–	<.0001*	1.0000	1.0000	–	–
3 Year	0.9 ± 0.2 (71)	0.9 ± 0.2 (72)	0.1534	<.0001*	1.0000	1.0000	1.0000	–	<.0001*	1.0000	1.0000	1.0000	–
5 Year	1.0 ± 0.1 (39)	0.9 ± 0.1 (34)	0.1259	<.0001*	<.0001*	1.0000	0.3516	1.0000	<.0001*	0.3336	1.0000	1.0000	1.0000
HHS													
Preop	50.3 ± 12.2 (113)	48.7 ± 13.5 (109)	0.2623	–	–	–	–	–	–	–	–	–	–
6 Month	93.0 ± 9.8 (100)	94.6 ± 7.7 (94)	0.2602	<.0001*	–	–	–	–	<.0001*	–	–	–	–
1 Year	96.1 ± 7.7 (85)	96.6 ± 5.5 (92)	0.8948	<.0001*	<.0001*	–	–	–	<.0001*	0.0418*	–	–	–
2 Year	96.5 ± 7.8 (72)	96.1 ± 6.5 (72)	0.1446	<.0001*	<.0001*	0.2509	–	–	<.0001*	0.0833	1.0000	–	–
3 Year	96.2 ± 8.3 (71)	97.6 ± 5.7 (71)	0.29	<.0001*	0.0003*	1.0000	1.0000	–	<.0001*	0.0106*	0.9757	0.2402	–
5 Year	98.4 ± 2.9 (39)	96.1 ± 5.9 (34)	0.0733	<.0001*	<.0001*	0.2266	0.3864	1.0000	<.0001*	0.0175*	1.0000	1.0000	1.0000
Oxford													
Preop	22.2 ± 7.4 (113)	21.9 ± 7.9 (109)	0.8968	–	–	–	–	–	–	–	–	–	–
6 Month	43.8 ± 5.4 (101)	42.9 ± 5.5 (94)	0.1147	<.0001*	–	–	–	–	<.0001*	–	–	–	–
1 Year	45.0 ± 5.4 (86)	44.6 ± 4.3 (92)	0.0776	<.0001*	0.0180*	–	–	–	<.0001*	0.0153*	–	–	–
2 Year	45.5 ± 4.6 (73)	45.2 ± 3.9 (72)	0.1347	<.0001*	0.0001*	1.0000	–	–	<.0001*	0.0039*	0.4147	–	–
3 Year	45.7 ± 4.8 (71)	45.9 ± 3.2 (71)	0.1796	<.0001*	0.0024*	1.0000	1.0000	–	<.0001*	0.0003*	0.0871	1.0000	–
5 Year	46.8 ± 1.9 (42)	46.0 ± 3.3 (34)	0.2548	<.0001*	<.0001*	0.4758	1.0000	1.0000	<.0001*	<.0001*	1.0000	1.0000	1.0000
Aluminum (μg/L)													
Preop	12.3 ± 1.9 (21)	11.6 ± 1.7 (10)	0.183	–	–	–	–	–	–	–	–	–	–
6 Month	12.3 ± 1.8 (16)	11.9 ± 1.8 (9)	0.5903	1.0000	–	–	–	–	1.0000	–	–	–	–
1 Year	13.9 ± 3.2 (13)	14.5 ± 4.2 (11)	0.7495	1.0000	1.0000	–	–	–	0.8438	1.0000	–	–	–
2 Year	13.3 ± 2.0 (12)	14.4 ± 5.3 (9)	0.2267	1.0000	1.0000	1.0000	–	–	1.0000	1.0000	1.0000	–	–
5 Year	14.7 ± 3.1 (4)	13.4 ± 1.5 (3)	0.2888	1.0000	1.0000	1.0000	–	1.0000	1.0000	1.0000	1.0000	–	1.0000
Titanium (μg/L)													
Preop	2.1 ± 0.6 (4)	1.2 ± 0.9 (5)	0.6242	–	–	–	–	–	–	–	–	–	–
6 Month	2.1 ± 1.3 (29)	2.4 ± 2.3 (14)	0.1859	1.0000	–	–	–	–	1.0000	–	–	–	–
1 Year	2.0 ± 1.1 (20)	2.4 ± 2.3 (15)	0.137	1.0000	1.0000	–	–	–	1.0000	1.0000	–	–	–
2 Year	2.2 ± 0.8 (15)	2.4 ± 3.4 (12)	0.0667	1.0000	1.0000	1.0000	–	–	1.0000	1.0000	1.0000	–	–
5 Year	1.3 ± 1.3 (3)	1.2 ± 0.2 (2)	1.0000	1.0000	1.0000	1.0000	–	1.0000	1.0000	1.0000	1.0000	–	1.0000

**Table A.7.** Subgroup analysis by primary diagnosis.

Follow up visit	Mean value and comparison within time period			Comparison by time within subgroups (Bonferroni adjusted *p*-value)									
	Osteoarthritis	Other diagnosis		Osteoarthritis					Other diagnosis				
	Unadjusted mean ± SD (*n*)	Unadjusted mean ± SD (*n*)	*p*-value	Preop	6 Month	1 Year	2 Year	3 Year	Preop	6 Month	1 Year	2 Year	3 Year
UCLA													
Preop	4.0 ± 1.6 (179)	3.4 ± 1.3 (43)	0.0253*	–	–	–	–	–	–	–	–	–	–
6 Month	5.5 ± 1.4 (155)	5.6 ± 1.5 (40)	0.8327	<.0001*	–	–	–	–	<.0001*	–	–	–	–
1 Year	5.8 ± 1.6 (140)	6.2 ± 1.6 (38)	0.1037	<.0001*	0.1099	–	–	–	<.0001*	0.4049	–	–	–
2 Year	5.9 ± 1.7 (115)	6.6 ± 1.2 (30)	0.0453*	<.0001*	0.0024*	1.0000	–	–	<.0001*	0.1472	1.0000	–	–
3 Year	6.2 ± 1.6 (112)	6.3 ± 1.5 (31)	0.7259	<.0001*	<.0001*	1.0000	1.0000	–	<.0001*	1.0000	1.0000	1.0000	–
5 Year	6.5 ± 1.5 (24)	6.3 ± 1.4 (52)	0.8108	<.0001*	0.0069*	1.0000	1.0000	1.0000	<.0001*	1.0000	1.0000	1.0000	1.0000
EQ5D													
Preop	0.5 ± 0.3 (179)	0.3 ± 0.3 (43)	0.0001*	–	–	–	–	–	–	–	–	–	–
6 Month	0.9 ± 0.1 (154)	0.8 ± 0.2 (39)	0.4058	<.0001*	–	–	–	–	<.0001*	–	–	–	–
1 Year	0.9 ± 0.1 (138)	0.9 ± 0.2 (38)	0.3308	<.0001*	0.1244	–	–	–	<.0001*	0.8327	–	–	–
2 Year	0.9 ± 0.2 (114)	0.9 ± 0.1 (30)	0.4594	<.0001*	1.0000	1.0000	–	–	<.0001*	1.0000	1.0000	–	–
3 Year	0.9 ± 0.2 (112)	0.9 ± 0.2 (31)	0.4253	<.0001*	1.0000	1.0000	1.0000	–	<.0001*	1.0000	1.0000	1.0000	–
5 Year	1.0 ± 0.1 (50)	0.9 ± 0.1 (23)	0.5801	<.0001*	0.0001*	1.0000	0.4633	1.0000	<.0001*	0.6079	1.0000	1.0000	1.0000
HHS													
6 Month	94.5 ± 8.3 (155)	90.9 ± 10.6 (39)	0.0198*	<.0001*	–	–	–	–	<.0001*	–	–	–	–
1 Year	96.4 ± 6.8 (139)	96.0 ± 6.3 (38)	0.1746	<.0001*	<.0001*	–	–	–	<.0001*	<.0001*	–	–	–
2 Year	96.2 ± 7.7 (114)	96.9 ± 4.6 (30)	0.9525	<.0001*	<.0001*	1.0000	–	–	<.0001*	0.0002*	1.0000	–	–
3 Year	96.8 ± 7.4 (111)	97.3 ± 6.2 (31)	0.9332	<.0001*	0.0010*	1.0000	1.0000	–	<.0001*	0.0003*	1.0000	0.6042	–
5 Year	97.2 ± 4.8 (50)	97.5 ± 4.517 (23)	0.8771	<.0001*	<.0001*	1.0000	1.0000	1.0000	<.0001*	0.0136*	1.0000	1.0000	1.0000
Preop	51.0 ± 12.4 (179)	43.5 ± 13.1 (43)	0.0012*	–	–	–	–	–	–	–	–	–	–
Oxford													
Preop	22.9 ± 7.4 (179)	18.7 ± 7.6 (43)	0.0020*	–	–	–	–	–	–	–	–	–	–
6 Month	43.8 ± 5.1 (155)	41.6 ± 6.3 (40)	0.0419*	<.0001*	–	–	–	–	<.0001*	–	–	–	–
1 Year	44.8 ± 4.8 (140)	45.0 ± 4.8 (38)	0.8912	<.0001*	0.0551*	–	–	–	<.0001*	0.0012*	–	–	–
2 Year	45.1 ± 4.6 (115)	46.3 ± 2.6 (30)	0.3042	<.0001*	<.0001*	0.2906	–	–	<.0001*	0.0017*	1.0000	–	–
3 Year	45.5 ± 4.3 (111)	47.0 ± 2.6 (31)	0.0398*	<.0001*	0.0008*	0.5435	1.0000	–	<.0001*	<.0001*	0.2233	0.1721	–
5 Year	46.2 ± 2.5 (52)	46.8 ± 2.9 (24)	0.1114	<.0001*	<.0001*	1.0000	1.0000	1.0000	<.0001*	0.0005*	1.0000	0.4468	1.0000
Aluminum (μg/L)													
Preop	12.0 ± 1.8 (29)	13.8 ± 0.6 (2)	0.0643	–	–	–	–	–	–	–	–	–	–
6 Month	12.1 ± 1.8 (22)	12.9 ± 1.8 (3)	0.4264	1.0000	–	–	–	–	1.0000	–	–	–	–
1 Year	14.5 ± 3.8 (21)	12.3 ± 2.3 (3)	0.2377	0.3418	0.3906	–	–	–	1.0000	1.0000	–	–	–
2 Year	13.4 ± 3.8 (18)	16.0 ± 2.1 (3)	0.056	1.0000	1.0000	0.6738	–	–	1.0000	1.0000	1.0000	–	–
5 Year	14.0 ± 2.3 (5)	14.5 ± 4.0 (2)	0.6985	1.0000	1.0000	1.0000	–	1.0000	1.0000	1.0000	1.0000	–	1.0000
Titanium (μg/L)													
Preop	2.1 ± 0.7 (8)	2.8 ± 0 (1)	0.4386	–	–	–	–	–	–	–	–	–	–
6 Month	2.4 ± 1.7 (37)	2.2 ± 2.1 (6)	0.0795	0.625	–	–	–	–	1.0000	–	–	–	–
1 Year	2.5 ± 1.8 (29)	2.2 ± 1.9 (6)	0.2361	0.625	1.0000	–	–	–	1.0000	1.0000	–	–	–
2 Year	2.9 ± 2.5 (24)	3.1 ± 1.9 (3)	0.4626	0.9375	1.0000	1.0000	–	–	1.0000	1.0000	1.0000	–	–
5 Year	1.3 ± 0.3 (4)	1.3 ± 0 (1)	1.0000	1.0000	1.0000	1.0000	–	1.0000	1.0000	1.0000	1.0000	–	1.0000

**Table A.8.** Subgroup analysis by tobacco consumption.

Follow up visit	Mean value and comparison within time period			Comparison by time within subgroups (Bonferroni adjusted *p*-value)									
	Smokers	Non smokers		Smokers					Non smokers				
	Unadjusted mean ± SD (*n*)	Unadjusted mean ± SD (*n*)	*p*-value	Preop	6 Month	1 Year	2 Year	3 Year	Preop	6 Month	1 Year	2 Year	3 Year
UCLA													
Preop	3.9 ± 1.4 (26)	3.8 ± 1.6 (196)	0.7261	–	–	–	–	–	–	–	–	–	–
6 Month	5.0 ± 1.2 (23)	5.6 ± 1.4 (172)	0.0699	0.0042*	–	–	–	–	<.0001*	–	–	–	–
1 Year	6.2 ± 1.8 (17)	5.9 ± 1.6 (161)	0.4296	0.0018*	1.0000	–	–	–	<.0001*	0.0653	–	–	–
2 Year	6.2 ± 1.7 (16)	6.1 ± 1.6 (129)	0.8755	0.0110*	0.8057	1.0000	–	–	<.0001*	0.0004*	1.0000	–	–
3 Year	6.0 ± 1.5 (16)	6.2 ± 1.6 (127)	0.5333	0.0037*	1.0000	1.0000	1.0000	–	<.0001*	0.0002*	1.0000	1.0000	–
5 Year	6.4 ± 0.9 (5)	6.5 ± 1.5 (71)	0.8782	0.0042*	0.2710	1.0000	1.0000	1.0000	<.0001*	0.0258*	1.0000	1.0000	1.0000
EQ5D													
Preop	0.4 ± 0.3 (26)	0.5 ± 0.3 (196)	0.2153	–	–	–	–	–	–	–	–	–	–
6 Month	0.9 ± 0.1 (23)	0.9 ± 0.2 (170)	0.4689	<.0001*	–	–	–	–	<.0001*	–	–	–	–
1 Year	1.0 ± 0.1 (17)	0.9 ± 0.2 (159)	0.1436	0.0002*	0.2344	–	–	–	<.0001*	0.0852	–	–	–
2 Year	0.9 ± 0.3 (16)	0.9 ± 0.1 (128)	0.7519	0.0023*	1.0000	1.0000	–	–	<.0001*	0.1393	1.0000	–	–
3 Year	0.9 ± 0.2 (16)	0.9 ± 0.2 (127)	0.314	0.0005*	1.0000	1.0000	1.0000	–	<.0001*	1.0000	1.0000	1.0000	–
5 Year	1.0 ± 0.0 (5)	0.9 ± 0.1 (68)	0.2101	0.9375	1.0000	1.0000	1.0000	1.0000	<.0001*	0.0001*	1.0000	0.7060	1.0000
HHS													
Preop	49.2 ± 11.8 (26)	49.5 ± 13.0 (196)	0.8863	–	–	–	–	–	–	–	–	–	–
6 Month	97.4 ± 4.3 (23)	93.3 ± 9.2 (171)	0.0289*	<.0001*	–	–	–	–	<.0001*	–	–	–	–
1 Year	98.5 ± 3.1 (17)	96.1 ± 6.9 (160)	0.1225	0.0002*	1.0000	–	–	–	<.0001*	<.0001*	–	–	–
2 Year	98.3 ± 3.6 (16)	96.1 ± 7.4 (128)	0.207	0.0005*	1.0000	1.0000	–	–	<.0001*	<.0001*	1.0000	–	–
3 Year	98.2 ± 2.6 (16)	96.8 ± 7.5 (126)	0.5922	0.0005*	1.0000	1.0000	1.0000	–	<.0001*	<.0001*	1.0000	1.0000	–
5 Year	99.0 ± 1.4 (5)	97.2 ± 4.8 (68)	0.6121	0.9375	1.0000	1.0000	1.0000	1.0000	<.0001*	<.0001*	1.0000	0.3798	1.0000
Oxford													
Preop	20.7 ± 6.2 (26)	22.2 ± 7.8 (196)	0.2549	–	–	–	–	–	–	–	–	–	–
6 Month	44.7 ± 4.1 (23)	43.2 ± 5.6 (172)	0.2523	<.0001*	–	–	–	–	<.0001*	–	–	–	–
1 Year	47.0 ± 1.7 (17)	44.6 ± 5.0 (161)	0.0420*	0.0002*	0.0073*	–	–	–	<.0001*	0.0021*	–	–	–
2 Year	45.9 ± 3.7 (16)	45.3 ± 4.3 (129)	0.4761	0.0005*	1.0000	1.0000	–	–	<.0001*	<.0001*	0.0698	–	–
3 Year	46.9 ± 1.6 (16)	45.7 ± 4.2 (126)	0.5381	0.0005*	0.6152	1.0000	1.0000	–	<.0001*	<.0001*	0.0293*	1.0000	–
5 Year	47.2 ± 1.3 (5)	46.4 ± 2.7 (71)	0.5873	0.9375	1.0000	1.0000	1.0000	1.0000	<.0001*	<.0001*	0.7167	1.0000	1.0000
Aluminum (μg/L)													
Preop	12.7 ± 1.8 (3)	12.0 ± 1.9 (28)	0.3002	–	–	–	–	–	–	–	–	–	–
6 Month	11.01.8 (5)	12.5 ± 1.8 (20)	0.6434	1.0000	–	–	–	–	1.0000	–	–	–	–
1 Year	14.1 ± 3.8 (5)	14.2 ± 4.0 (19)	0.2143	1.0000	0.75	–	–	–	0.1855	1.0000	–	–	–
2 Year	12.3 ± 3.8 (4)	14.1 ± 4.0 (17)	0.5015	1.0000	1.0000	1.0000	–	–	1.0000	1.0000	1.0000	–	–
5 Year	(0)	14.1 ± 2.5 (7)	–	–	–	–	–	–	1.0000	1.0000	1.0000	–	1.0000
Titanium (μg/L)													
Preop	1.1 ± 0 (1)	2.3 ± 0.6 (8)	0.1213	–	–	–	–	–	–	–	–	–	–
6 Month	1.9 ± 1.0 (10)	2.5 ± 1.9 (33)	0.3071	1.0000	–	–	–	–	1.0000	–	–	–	–
1 Year	4.5 ± 2.3 (5)	2.2 ± 1.5 (30)	0.0376*	1.0000	0. 6250	–	–	–	0.6250	1.0000	–	–	–
2 Year	3.3 ± 0.6 (5)	2.8 ± 2.7 (22)	0.0243*	1.0000	1.0000	1.0000	–	–	1.0000	1.0000	1.0000	–	–
5 Year	(0)	1.3 ± 0.3 (5)	–	–	–	–	–	–	1.0000	1.0000	1.0000	–	1.0000

**Table A.9.** Subgroup analysis by alcohol consumption.

Follow up visit	Mean value and comparison within time period			Comparison by time within subgroups (Bonferroni adjusted *p*-value)									
	Alcohol consumers	Non alcohol consumers		Alcohol consumers					Non alcohol consumers				
	Unadjusted mean ± SD (*n*)	Unadjusted mean ± SD (*n*)	*p*-value	Preop	6 Month	1 Year	2 Year	3 Year	Preop	6 Month	1 Year	2 Year	3 Year
UCLA													
Preop	4.5 ± 1.7 (73)	3.5 ± 1.5 (149)	<.0001*	–	–	–	–	–	–	–	–	–	–
6 Month	5.6 ± 1.4 (65)	5.5 ± 1.4 (130)	0.4434	<.0001*	–	–	–	–	<.0001*	–	–	–	–
1 Year	6.2 ± 1.6 (64)	5.7 ± 1.6 (114)	0.0064*	<.0001*	0.0130*	–	–	–	<.0001*	0.9955	–	–	–
2 Year	6.5 ± 1.6 (54)	5.8 ± 1.6 (91)	0.0125*	<.0001*	0.0001*	1.0000	–	–	<.0001*	0.2088	1.0000	–	–
3 Year	6.4 ± 1.5 (54)	6.1 ± 1.7 (89)	0.1955	<.0001*	0.0014*	1.0000	1.0000	–	<.0001*	0.0187*	1.0000	1.0000	–
5 Year	7.0 ± 1.3 (28)	6.1 ± 1.4 (48)	0.0195*	<.0001*	0.0020*	0.9229	1.0000	1.0000	<.0001*	1.0000	1.0000	1.0000	1.0000
EQ5D													
Preop	0.4 ± 0.3 (73)	0.5 ± 0.3 (149)	0.634	–	–	–	–	–	–	–	–	–	–
6 Month	0.9 ± 0.2 (64)	0.9 ± 0.2 (129)	0.4121	<.0001*	–	–	–	–	<.0001*	–	–	–	–
1 Year	0.9 ± 0.1 (63)	0.9 ± 0.2 (113)	0.9971	<.0001*	0.0396*	–	–	–	<.0001*	0.6517	–	–	–
2 Year	0.9 ± 0.2 (54)	0.9 ± 0.1 (90)	0.1211	<.0001*	0.1935	1.0000	–	–	<.0001*	1.0000	1.0000	–	–
3 Year	0.9 ± 0.2 (54)	0.9 ± 0.2 (89)	0.708	<.0001*	1.0000	1.0000	1.0000	–	<.0001*	1.0000	1.0000	1.0000	–
5 Year	1.0 ± 0.1 (27)	0.9 ± 0.1 (46)	0.2379	<.0001*	0.0879	1.0000	1.0000	1.0000	<.0001*	0.0031*	1.0000	0.8459	1.0000
HHS													
Preop	51.9 ± 13.7 (73)	48.3 ± 12.3 (149)	0.0638	–	–	–	–	–	–	–	–	–	–
6 Month	92.6 ± 7.9 (64)	94.4 ± 9.3 (130)	0.0091*	<.0001*	–	–	–	–	<.0001*	–	–	–	–
1 Year	96.0 ± 7.4 (64)	96.5 ± 6.2 (113)	0.6825	<.0001*	0.0005*	–	–	–	<.0001*	<.0001*	–	–	–
2 Year	97.2 ± 6.2 (54)	95.8 ± 7.6 (90)	0.0749	<.0001*	<.0001*	1.0000	–	–	<.0001*	0.0024*	1.0000	–	–
3 Year	97.2 ± 6.9 (53)	96.7 ± 7.3 (89)	0.832	<.0001*	<.0001*	1.0000	1.0000	–	<.0001*	0.0430*	1.0000	1.0000	–
5 Year	97.9 ± 3.5 (28)	97.0 ± 5.3 (45)	0.7873	<.0001*	<.0001*	1.0000	1.0000	1.0000	<.0001*	0.0001*	1.0000	0.6599	1.0000
Oxford													
Preop	23.3 ± 7.3 (73)	21.4 ± 7.7 (149)	0.1289	–	–	–	–	–	–	–	–	–	–
6 Month	42.4 ± 5.1 (64)	43.8 ± 5.6 (131)	0.0017*	<.0001*	–	–	–	–	<.0001*	–	–	–	–
1 Year	44.8 ± 4.9 (64)	44.8 ± 4.8 (114)	0.7546	<.0001*	<.0001*	–	–	–	<.0001*	0.2514	–	–	–
2 Year	45.6 ± 3.3 (54)	45.2 ± 4.7 (91)	0.9398	<.0001*	<.0001*	0.1791	–	–	<.0001*	0.0371*	1.0000	–	–
3 Year	45.9 ± 3.8 (54)	45.7 ± 4.2 (88)	0.3502	<.0001*	<.0001*	0.1373	1.0000	–	<.0001*	0.0399*	0.9436	1.0000	–
5 Year	46.7 ± 2.4 (28)	46.3 ± 2.8 (48)	0.2696	<.0001*	<.0001*	0.6017	1.0000	1.0000	<.0001*	<.0001*	1.0000	1.0000	1.0000
Aluminum (μg/L)													
Preop	11.0 ± 0.6 (2)	12.2 ± 1.9 (29)	0.2957	–	–	–	–	–	–	–	–	–	–
6 Month	(0)	12.2 ± 1.8 (25)	–	–	–	–	–	–	1.0000	–	–	–	–
1 Year	(0)	14.2 ± 3.6 (24)	–	–	–	–	–	–	0.3418	1.0000	–	–	–
2 Year	12.7 ± 1.4 (2)	13.9 ± 3.8 (19)	0.9522	1.0000	–	–	–	–	1.0000	1.0000	1.0000	–	–
5 Year	(0)	14.1 ± 2.5 (7)	–	–	–	–	–	–	1.0000	1.0000	1.0000	–	1.0000
Titanium (μg/L)													
Preop	(0)	2.1 ± 0.7 (9)	–	–	–	–	–	–	–	–	–	–	–
6 Month	(0)	2.4 ± 1.7 (43)	–	–	–	–	–	–	0.625	–	–	–	–
1 Year	6.0 ± 0 (1)	2.4 ± 1.7 (34)	0.1646	–	–	–	–	–	0.625	1.0000	–	–	–
2 Year	3.1 ± 0.8 (2)	2.9 ± 2.5 (25)	0.3075	–	–	1.0000	–	–	0.9375	1.0000	1.0000	–	–
5 Year	(0)	1.3 ± 0.3 (5)	–	–	–	–	–	–	1.0000	1.0000	1.0000	–	1.0000

**Table A.10.** Subgroup analysis by femoral size.

Follow up visit	Mean value and comparison within time period			Comparison by time within subgroups (Bonferroni adjusted *p*-value)									
	<10	≥10		<10					≥10				
	Unadjusted mean ± SD (*n*)	Unadjusted mean ± SD (*n*)	*p*-value	Preop	6 Month	1 Year	2 Year	3 Year	Preop	6 Month	1 Year	2 Year	3 Year
UCLA													
Preop	3.5 ± 1.3 (93)	4.1 ± 1.7 (129)	0.0060*	−	−	−	−	−	−	−	−	−	−
6 Month	5.6 ± 1.4 (84)	5.5 ± 1.4 (111)	0.4442	<.0001*	−	−	−	−	<.0001*	−	−	−	−
1 Year	6.2 ± 1.5 (72)	5.7 ± 1.6 (106)	0.0741	<.0001*	0.0039*	−	−	−	<.0001*	1.0000	−	−	−
2 Year	6.2 ± 1.5 (64)	6.0 ± 1.7 (81)	0.4584	<.0001*	0.0163*	1.0000	–	–	<.0001*	0.0111*	1.0000	−	−
3 Year	6.3 ± 1.4 (62)	6.1 ± 1.7 (81)	0.3142	<.0001*	0.0098*	1.0000	1.0000	–	<.0001*	0.0034*	1.0000	1.0000	−
5 Year	6.5 ± 1.4 (44)	6.3 ± 1.5 (32)	0.5344	<.0001*	0.1310	1.0000	1.0000	1.0000	<.0001*	0.2771	1.0000	1.0000	1.0000
EQ5D													
Preop	0.4 ± 0.3 (93)	0.5 ± 0.3 (129)	0.1245	−	−	−	−	−	−	−	−	−	−
6 Month	0.9 ± 0.2 (81)	0.9 ± 0.2 (112)	0.3409	<.0001*	−	−	−	−	<.0001*	−	−	−	−
1 Year	0.9 ± 0.2 (71)	0.9 ± 0.2 (105)	0.9195	<.0001*	0.0072*	−	−	−	<.0001*	1.0000	−	−	−
2 Year	0.9 ± 0.2 (64)	0.9 ± 0. (80)	0.1061	<.0001*	1.0000	0.6837	−	−	<.0001*	1.0000	1.0000	−	−
3 Year	0.9 ± 0.1 (62)	0.9 ± 0.2 (81)	0.9483	<.0001*	0.5022	1.0000	1.0000	–	<.0001*	1.0000	1.0000	1.0000	−
5 Year	1.0 ± 0.1 (41)	0.9 ± 0.1 (32)	0.1239	<.0001*	0.0002*	0.5475	0.1208	1.0000−	<.0001*	0.3853	1.0000	1.0000	1.0000
HHS													
Preop	47.9 ± 13.1 (93)	50.7 ± 12.6 (129)	0.1137	−	−	−	−	−	−	−	−	−	−
6 Month	93.0 ± 9.2 (82)	94.3 ± 8.6 (112)	0.2306	<.0001*	−	−	−	−	<.0001*	−	−	−	−
1 Year	95.7 ± 6.3 (71)	96.8 ± 6.9 (106)	0.0074*	<.0001*	0.0002*	−	−	−	<.0001*	<.0001*	−	−	−
2 Year	96.3 ± 5.8 (63)	96.4 ± 8.1 (81)	0.0845	<.0001*	0.0001*	1.0000	−	−	<.0001*	0.0003*	1.0000	–	−
3 Year	98.5 ± 2.7 (61)	95.7 ± 8.9 (81)	0.3012	<.0001*	<.0001*	0.0007*	0.0053*	–	<.0001*	0.1254	1.0000	1.0000	−
5 Year	97.7 ± 3.8 (42)	96.7 ± 5.7 (31)	0.6720	<.0001*	<.0001*	0.0456*	0.2106	1.0000	<.0001*	0.0356*	1.0000	1.0000	1.0000
Oxford													
Preop	21.4 ± 7.6 (93)	22.6 ± 7.7 (129)	0.5275	–	–	–	–	–	–	–	–	–	–
6 Month	42.6 ± 6.0 (83)	44.0 ± 5.0 (112)	0.0411*	<.0001*	–	–	–	–	<.0001*	–	–	–	−
1 Year	44.9 ± 5.0 (72)	44.7 ± 4.7 (106)	0.8265	<.0001*	<.0001*	−	−	−	<.0001*	0.3877	–	–	−
2 Year	45.5 ± 3.3 (64)	45.2 ± 4.9 (81)	0.4259	<.0001*	<.0001*	1.0000	–	–	<.0001*	0.0199*	0.3371	–	−
3 Year	46.6 ± 2.4 (61)	45.2 ± 4.8 (81)	0.1934	<.0001*	<.0001*	0.0128*	0.0124*	–	<.0001*	0.0442*	1.0000	1.0000	−
5 Year	46.8 ± 2.0 (44)	45.9 ± 3.3 (32)	0.1677	<.0001*	<.0001*	0.0734	0.8583	1.0000	<.0001*	0.0022*	1.0000	1.0000	1.0000
Aluminum (μg/L)													
Preop	12.1 ± 2.5 (10)	12.1 ± 1.5 (21)	0.5826	–	–	–	–	–	–	–	–	–	–
6 Month	11.6 ± 1.8 (9)	12.5 ± 1.7 (16)	0.1565	1.0000	–	–	–	–	1.0000	–	–	–	–
1 Year	16.6 ± 5.3 (6)	13.4 ± 2.6 (18)	0.1709	1.0000	1.0000	–	–	–	1.0000	1.0000	–	–	–
2 Year	13.4 ± 3.0 (6)	13.9 ± 4.0 (15)	0.6402	1.0000	1.0000	1.0000	–	–	1.0000	1.0000	1.0000	–	–
5 Year	14.4 ± 0 (1)	14.1 ± 2.7 (6)	1.0000	1.0000	1.0000	1.0000	–	1.0000	1.0000	1.0000	1.0000	–	1.0000
Titanium (μg/L)													
Preop	1.7 ± 1.7 (4)	2.5 ± 0.6 (5)	0.05	−	−	−	−	−	−	−	−	−	–
6 Month	2.7 ± 2.7 (16)	2.2 ± 1.4 (27)	0.6967	1.0000	−	−	−	−	1.0000	−	−	−	–
1 Year	2.8 ± 2.8 (10)	2.4 ± 1.6 (25)	0.3412	1.0000	1.0000	−	−	−	1.0000	1.0000	−	−	–
2 Year	2.9 ± 2.9 (8)	2.9 ± 2.9 (19)	0.2638	1.0000	1.0000	1.0000	–	–	1.0000	1.0000	1.0000	−	–
5 Year	0	1.3 ± 0.3 (5)	−	−	−	−	−	−	1.0000	1.0000	1.0000	−	1.0000

**Table A.11.** Group analysis by previous surgery.

Follow up visit	Mean value and comparison within time period			Comparison by time within subgroups (Bonferroni adjusted *p*-value)									
	Previous surgery	No previous surgery		Previous surgery					No previous surgery				
	Unadjusted mean ± SD (*n*)	Unadjusted mean ± SD (*n*)	*p*-value	Preop	6 Month	1 Year	2 Year	3 Year	Preop	6 Month	1 Year	2 Year	3 Year
UCLA													
Preop	4.1 ± 1.4 (55)	3.8 ± 1.7 (167)	0.0400*	−	−	−	−	−	−	−	−	−	−
6 Month	5.6 ± 1.2 (49)	5.5 ± 1.5 (146)	0.5119	<.0001*	−	−	−	−	<.0001*	−	−	−	−
1 Year	6.0 ± 1.6 (42)	5.9 ± 1.6 (136)	0.6423	<.0001*	1.0000	−	−	−	<.0001*	0.0336*	−	−	−
2 Year	6.3 ± 1.8 (34)	6.0 ± 1.6 (111)	0.2617	<.0001*	0.1756	1.0000	−	−	<.0001*	0.0013*	1.0000	−	−
3 Year	6.2 ± 1.7 (31)	6.2 ± 1.6 (112)	0.8897	<.0001*	0.4093	1.0000	1.0000	−	<.0001*	0.0001*	1.0000	1.0000	−
5 Year	6.6 ± 1.3 (18)	6.4 ± 1.5 (58)	0.8182	0.0001*	1.0000	0.9229	1.0000	1.0000	<.0001*	0.0089*	1.0000	1.0000	1.0000
EQ5D													
Preop	0.4 ± 0.3 (55)	0.5 ± 0.3 (167)	0.7091	−	−	−	−	−	−	−	−	−	−
6 Month	0.9 ± 0.1 (49)	0.9 ± 0.2 (144)	0.6602	<.0001*	−	−	−	−	<.0001*	−	−	−	−
1 Year	0.9 ± 0.2 (41)	0.9 ± 0.1 (135)	0.2375	<.0001*	1.0000	−	−	−	<.0001*	0.0061*	−	−	−
2 Year	0.9 ± 0.2 (34)	0.9 ± 0.2 (110)	0.815	<.0001*	1.0000	1.0000	−	−	<.0001*	0.0838	1.0000	−	−
3 Year	0.9 ± 0.2 (31)	0.9 ± 0.2 (112)	0.2485	<.0001*	1.0000	1.0000	1.0000	−	<.0001*	0.0832	1.0000	1.0000	−
5 Year	1.0 ± 0.1 (17)	0.9 ± 0.1 (56)	0.4700	0.0002*	0.0586	1.0000	1.0000	1.0000	<.0001*	0.0013*	1.0000	1.0000	1.0000
HHS													
Preop	49.4 ± 11.7 (55)	49.5 ± 13.3 (167)	0.5833	−	−	−	−	−	−	−	−	−	−
6 Month	93.2 ± 9.1 (49)	94.0 ± 8.8 (145)	0.44	−	−	−	−	−	<.0001*	−	−	−	−
1 Year	95.9 ± 7.5 (42)	96.5 ± 6.4 (135)	0.7247	<.0001*	0.0005*	−	−	−	<.0001*	<.0001*	−	−	−
2 Year	96.2 ± 6.7 (34)	96.4 ± 7.3 (110)	0.2937	<.0001*	0.0035*	0.0403	–	–	<.0001*	<.0001*	1.0000	–	–
3 Year	95.1 ± 10.4 (31)	97.4 ± 5.8 (111)	0.4157	<.0001*	0.0251*	1.0000	1.0000	–	<.0001*	<.0001*	0.9966	0.5599	–
5 Year	97.6 ± 3.7 (16)	97.2±4.9 (57)	0.9699	<.0001*	0.9577	1.0000	1.0000	1.0000	<.0001*	<.0001*	0.6425	0.2189	1.0000
Oxford													
Preop	23.0 ± 7.9 (55)	21.8 ± 7.5 (167)	0.3217	−	−	−	−	−	−	−	−	−	−
6 Month	43.8 ± 5.3 (49)	43.2 ± 5.3 (146)	0.3921	<.0001*	−	−	−	−	<.0001*	−	−	−	−
1 Year	43.7 ± 4.2 (42)	45.1 ± 4.2 (136)	0.1258	<.0001*	0.0001*	−	−	−	<.0001*	<.0001*	−	−	−
2 Year	45.4 ± 4.3 (34)	45.3 ± 4.3 (111)	0.9098	<.0001*	1.0000	0.0037*	–	–	<.0001*	<.0001*	1.0000	−	−
3 Year	44.9 ± 3.2 (31)	46.1 ± 3.2 (111)	0.8579	<.0001*	0.214	0.1455	1.0000	–	<.0001*	<.0001*	0.0674	0.4474	–
5 Year	47.0 ± 2.9 (18)	46.2 ± 2.9 (58)	0.3734	<.0001*	0.5892	1.0000	1.0000	1.0000	<.0001*	<.0001*	1.0000	1.0000	1.0000
Aluminum (μg/L)													
Preop	13.6 ± 3.1 (5)	11.8 ± 1.4 (26)	0.1469	−	−	−	−	−	−	−	−	−	−
6 Month	12.5 ± 2.0 (7)	12.1 ± 1.7 (18)	0.6065	1.0000	−	−	−	−	1.0000	−	−	−	−
1 Year	18.8 ± 6.8 (3)	13.5 ± 2.7 (21)	0.0881	1.0000	−	−	−	−	1.0000	1.0000	−	−	−
2 Year	16.0 ± 6.1 (5)	12.9 ± 1.8 (15)	0.7851	1.0000	1.0000	1.0000	–	–	1.0000	1.0000	1.0000	–	–
5 Year	14.1 ± 0 (1)	14.1 ± 2.7 (6)	0.6171	1.0000	1.0000	1.0000	–	1.0000	1.0000	1.0000	1.0000	–	1.0000
Titanium (μg/L)													
Preop	2.2 ± 0.7 (7)	2.1 ± 1.1 (2)	1.0000	–	–	–	–	–	–	–	–	–	–
6 Month	3.3 ± 2.0 (12)	2.0 ± 1.5 (31)	0.0006	1.0000	–	–	–	–	1.0000	–	–	–	–
1 Year	3.7 ± 2.4 (7)	2.2 ± 1.5 (28)	0.0131*	1.0000	1.0000	–	–	–	1.0000	1.0000	–	–	–
2 Year	3.9 ± 4.3 (8)	2.5 ± 1.0 (19)	0.632	1.0000	1.0000	1.0000	–	–	1.0000	1.0000	1.0000	–	–
5 Year	1.1 ± 0 (1)	1.4 ± 0.3 (4)	0.4795	1.0000	1.0000	1.0000	–	1.0000	1.0000	1.0000	1.0000	–	1.0000

**Table A.12.** Significant correlation between sociodemographic and surgical characteristics and surgical time, clinical scores and laboratory values.

Variables	Rho	*p*-value	*N*	
BMI	UCLA (2 Year)	−0.23	0.0056*	145
	TITANIUM (6 Month)	0.38	0.0132*	43
	TITANIUM (1 Year)	0.39	0.0210*	35
Age	UCLA (6 Month)	−0.20	0.0058*	195
	UCLA (1 Year)	−0.45	<.0001*	178
	UCLA (2 Year)	−0.46	<.0001*	145
	UCLA (3 Year)	−0.45	<.0001*	143
	UCLA (5 Year)	−0.39	0.0006*	76
	EQ5D (3 Year)	−0.24	0.0414*	73
	HHS (2 Year)	−0.17	0.0476*	144
	HHS (3 Year)	−0.21	0.0095*	142
	HHS (5 Year)	−0.31	0.0077*	73
	OXFORD (1 Year)	−0.21	0.0050*	178
	OXFORD (2 Year)	−0.22	0.0087*	145
	OXFORD (5 Year)	−0.26	0.0249*	76
Gender (female)	UCLA (Preop)	−0.21685	0.0011*	222
	UCLA (6 Month)	−0.21	0.0031*	195
	UCLA (5 Year)	−0.28	0.0132*	76
	EQ5D (Preop)	−0.21	0.0014*	222
	EQ5D (6 Month)	−0.18	0.0101*	193
	HHS (Preop)	−0.22	0.0009*	222
	HHS (6 Month)	−0.25	0.0005*	194
	HHS (1 Year)	−0.25	0.0009*	177
	HHS (2 Year)	−0.20	0.0149*	144
	OXFORD (Preop)	−0.25	0.0001*	222
	OXFORD (6 Month)	−0.25	0.0005*	195
	OXFORD (1 Year)	−0.15	0.0416*	178
	OXFORD (2 Year)	−0.23	0.0056*	145
Smoke	Surgical time	−0.15	0.0277*	222
	HHS (6 Month)	0.16	0.0285*	194
	OXFORD (1 Year)	0.15	0.0416*	178
	TITANIUM (1 Year)	0.37	0.0355*	35
	TITANIUM (2 Year)	0.44	0.0211*	27
Alcohol consumption	UCLA (Preop)	0.29	<.0001*	222
	UCLA (1 Year)	0.20	0.0061*	178
	UCLA (2 Year)	0.21	0.0120*	145
	UCLA (5 Year)	0.27	0.0185*	76
	HHS (6 Month)	−0.19	0.0088*	194
	OXFORD (6 Month)	−0.22	0.0016*	195
Previous surgery	UCLA (Preop)	0.14	0.0397*	222
	TITANIUM (6 Month)	0.53	0.0003*	43
	TITANIUM (1 Year)	0.43	0.0108*	35
Femoral size	UCLA (Preop)	0.15	0.0242*	222
	EQ5D (Preop)	0.14	0.0381*	222
	HHS (1 Year)	0.20	0.0081*	177
	OXFORD (6 Month)	0.22	0.0022*	195
	ALUMINM (6 Month)	0.40	0.0476*	25
	TITANIUM (Preop)	0.69	0.0399*	9
	TITANIUM (1 Year)	−0.36	0.0353*	35
Neck version: anteverted	EQ5D (3 Year)	−0.20	0.0163*	143
	HHS (6 Month)	−0.16	0.0231*	194
Neck version: straight	EQ5D (2 Year)	−0.17	0.0437*	144
Anterolateral surgical approach	UCLA (1 Year)	−0.22	0.0032*	178
	UCLA (2 Year)	−0.24	0.0034*	145
	UCLA (3 Year)	−0.25	0.0030*	143
	EQ5D (1 Year)	−0.15	0.0483*	176
	HHS (Preop)	−0.22	0.0012*	222
	TITANIUM (Preop)	0.87	0.0025*	9
	TITANIUM (2 Year)	−0.39	0.0445*	27
Direct lateral surgical approach	Surgical time	0.53	<.0001*	222
	EQ5D (Preop)	−0.13	0.0494*	222
	HHS (6 Month)	−0.22	0.0026*	194
	HHS (1 Year)	−0.18	0.0169*	177
	HHS (2 Year)	−0.23	0.0054*	144
	HHS (3 Year)	−0.28	0.0008*	142
	HHS (5 Year)	−0.29	0.0119*	73
	OXFORD (6 Month)	−0.18	0.0114*	195
Posterolateral surgical approach	Surgical time	−0.31	<.0001*	222
	UCLA (1 Year)	0.19	0.0104*	178
	UCLA (2 Year)	0.28	0.0006*	145
	UCLA (3 Year)	0.28	0.0007*	143
	UCLA (5 Year)	0.30	0.0087*	76
	HHS (Preop)	0.16	0.0171*	222
	HHS (5 Year)	0.25	0.0330*	73
	OXFORD (1 Year)	0.16	0.0312*	178
	OXFORD (3 Year)	0.17	0.0445*	142
	TITANIUMM (Preop)	−0.87	0.0025*	9
	TITANIUM (2 Year)	0.39	0.0445*	27
